# Model-based analysis of fatigued human knee extensors: Effects of isometrically induced fatigue on Hill-type model parameters and ballistic contractions

**DOI:** 10.1007/s00421-018-3875-2

**Published:** 2018-05-05

**Authors:** Harald Penasso, Sigrid Thaller

**Affiliations:** Institute of Sport Science, University of Graz, Mozartgasse 14, 8010 Graz, Austria

**Keywords:** Hill-type muscle model, Parameter identification, Activation dynamics, Force–velocity relation, Voluntary contraction, Fatigue

## Abstract

This study investigated the effect of isometrically induced fatigue on Hill-type muscle model parameters and related task-dependent effects. Parameter identification methods were used to extract fatigue-related parameter trends from isometric and ballistic dynamic maximum voluntary knee extensions. Nine subjects, who completed ten fatiguing sets, each consisting of nine 3 s isometric maximum voluntary contractions with 3 s rest plus two ballistic contractions with different loads, were analyzed. Only at the isometric task, the identified optimized model parameter values of muscle activation rate and maximum force generating capacity of the contractile element decreased from $20.8 \pm 8.4$ to $11.2 \pm 4.1$ Hz and from $18{,}137 \pm 150$ to $10{,}666 \pm 2139$ N, respectively. For all tasks, the maximum efficiency of the contractile element, mathematically related to the curvature of the force–velocity relation, increased from $0.35 \pm 0.04$ to $0.42 \pm 0.05$. The model parameter maximum contraction velocity decreased from $0.93 \pm 0.1$ to $0.9 \pm 0.1$ m/s and the stiffness of the serial elastic element from $1936 \pm 227$ to $1432 \pm 245$ N/mm. Thus, models of fatigue should consider fatigue dependencies in active as well as in passive elements, and muscle activation dynamics should account for the task dependency of fatigue.

## Introduction

Human movement is possible through the interaction of several structures including nervous system, muscles, tendons, and bones. As a consequence of high-intensity and long-duration contractions, interdependent systems of the body will react to the physical strain caused.

Since the early work of Mosso (1906), it is known that fatigue affects the central and peripheral systems simultaneously (Bigland-Ritchie et al. 1978; Enoka and Duchateau 2016; Gandevia 2001; Taylor et al. 2016). In voluntary movements, the fatigue-related changes in both systems cause the reversible reduction of measures of performance, e.g., power and force (Edman and Mattiazzi 1981; Hilty 2011; Williams and Ratel 2009). This is accompanied by an increased effort to maintain a sub-maximal task as well as by the sensation of exhaustion (Enoka and Stuart 1992; Noakes 2012). While the central system controls the level of voluntary activation, changes at or distal to the neuromuscular junction are ascribed to the peripheral system (Gandevia 2001). Furthermore, the magnitude and the effect of fatigue will depend on the modality of the movement, e.g., task dependency (Enoka and Stuart 1992; Taylor et al. 2016), contraction type (Babault et al. 2006; Griffin et al. 2000; Liu et al. 2003), contraction velocity (Harwood et al. 2011; Morel et al. 2015), and the level of achieved voluntary activation (Bigland-Ritchie et al. 1978; Gandevia et al. 1998; Sidhu et al. 2013).

The effects of fatigue on the interacting sub-structures of Hill-type muscle models, however, are not yet well studied. Models applied to fatiguing exercises should consider central as well as peripheral systems (Callahan et al. 2016; Hawkins and Hull 1993; Shorten et al. 2007). Central effects can be described by modeling the dynamics of motoneuron recruitment (Liu et al. 2003), and the peripheral force generation of the muscle–tendon complex (${\rm MTC}$) should consider changes at the contraction velocity-dependent contractile element (CE) (Jones et al. 2006) and the coupled serial elastic element (${\rm SEE}$) (Kubo et al. 2001).

The purpose of this study was to investigate the simultaneous effects of isometrically induced fatigue on Hill-type muscle model parameter values and muscle activation. Parameter identification tools were used to optimize model parameters within physiological limits to fit the measured force and position data. As some of the parameter values of activation dynamics, force–velocity relation (FV) and SEE were separately identified for isometric (ISO) and dynamic ballistic (BAL) maximum voluntary contractions (MVCs); also, task-dependent effects could be addressed.

## Methods

The subjects performed isometric and dynamic ballistic knee-extension MVCs at an instrumented inclined leg press and completed an intermitted isometric fatigue protocol. At each isometric MVC, the subjects were encouraged with a pre-recorded loud ‘3-2-1-go!’ sound (McNair et al. 1996). For all stages of fatigue, data from three contractions were simultaneously processed by a nonlinear parameter identification routine that fitted the knee-extension model output to the measured data (Penasso and Thaller 2017; Siebert et al. 2007).

### Measurements

#### Experimental setup

**Fig. 1:**
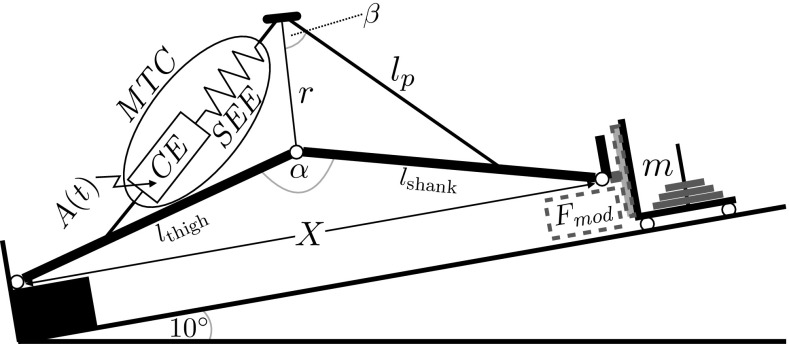
Sketch of the muscle–tendon complex (${\rm MTC}$) and of the geometric representation of the leg including the knee moment arm (*r*) as well as the lengths of thigh ($l_{\rm thigh}$), shank ($l_{\rm shank}$), and patellar tendon ($l_{\rm p}$) (proportions not in scale). Using the position (*X*) of the sledge (*m*), the knee-extension angle ($\alpha$) is calculated and $\beta$ is calculated numerically. In the model, a movement is initiated by activating the contractile element (CE) via activation dynamics (*A*(*t*)). Thus, the force of the ${\rm MTC}$, produced by the interaction of CE and serial elastic element (${\rm SEE}$), is transferred to the environment ($F_{{\rm mod}}$)

The subjects were seated and fastened to an inclined leg press (leg-press inclination 10$^\circ$; Tetra^®^ Ilmenau). The sledge of the leg press (32.5 kg + $2 \times 20$ kg additional load) could be allowed to slide or to be fixed at a desired position for isometric contractions and carried a force plate (Kistler type Z17068AA1; range $F_{x,y}$ 0–2.5 kN, $F_{z}$ 0–10 kN; sampling rate (SR) 500 Hz; Fig. 1). During ISO and BAL contractions, the sledge was positioned such that the initial knee-extension angle ($\alpha$) corresponded to 120$^\circ$ (cf. Fig. 1). To prevent additional force transmission from unwanted plantar flexion during the knee extensions, a 5 cm block of wood, used as a spacer, was mounted onto the force plate and served as heel contact. The sledge position was measured with a high-precision rotary encoder (Stegmann 4096 ppr, SR 500 Hz). Motion capture data obtained from reflective markers, placed at the greater trochanter, the lateral epicondyle of the knee, and the lateral malleolus (6 Vicon M2 cameras; SR 250 Hz), were used to correct subject displacements relative to the 1D leg-press coordinate system. Following Sust et al. (1997b), anthropometric measures of the thigh (greater trochanter to lateral knee joint line), the shank (lateral knee joint line to lateral malleolus), the knee circumference (CIR, measured at the height of the center of the patella to estimate the knee moment arm $r= {\rm CIR}/(2\pi )$), and the approximated length of the patellar tendon ($l_{\rm p}$, patella center to tuberositas tibiae, measured at the fully extended and relaxed leg) were taken with a tape measure (Fig. 1). Superficial muscle activities (sEMG) from the right m. vastus lateralis (VL), m. vastus medialis (VM), and m. rectus femoris (RF) were acquired according to SENIAM guidelines for bipolar configurations (myon 320; SR 4000 Hz) (Hermens et al. 2000). The activity of the deep vastus intermedius muscle was not measured. All data were synchronized and recorded on one measurement system (Dewetron).

#### Procedure

Subjects were familiarized with the testing procedure one week before the experiment.

**Fig. 2:**
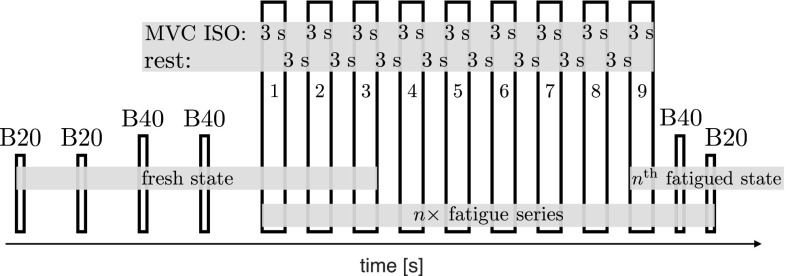
The measurement protocol started with two ballistic contractions against low load (B20, $32.5+20$ kg) and continued with two ballistic MVCs against higher load (B40, $32.5+40$ kg) separated by $\sim$ 10 s breaks. This was followed by the fatiguing protocol consisting of $n = 10$ identical sets. Each set included nine 3 s ISO which were separated by 3 s rest periods. Each 9th ISO was followed by two BALs [(1) B40, (2) B20]. The resting period prior to the BALs was about 5 s. Together with the manual unlocking and fixation of the sledge, the duration of one fatigue set was about 1 min. The preceding familiarization, preparation, and warm-up procedures are not shown

On the measurement day, the anthropometric measurements were taken, the sEMG electrodes as well as the reflective makers were placed and the subjects warmed up (5 min self-paced, self-rated low-intensity ergometer, 1 min severe ergometer exercise, 30 s crouching at 90$^\circ$ knee-angle, and ten squats). Subsequently, they sat down at the instrumented inclined leg press and were fastened to the seat. The measurement started with two ballistic MVCs against low loads (B20, $32.5+20$ kg) and continued with two BALs against higher loads (B40, $32.5+40$ kg; Fig. 2). Between these short contractions, the subjects recovered for about 10 s.

The initial procedure was followed by the fatiguing protocol consisting of ten identical sets (Fig. 2). Thereby, the number of fatiguing sets was not revealed to the subjects who agreed to go all out at each contraction. Each set included nine 3 s ISO which were separated by 3 s rest periods. The ISO tasks were automatically initiated and terminated by a pacing-trigger signal that played the 3 s long ‘3-2-1-go!’ sound. The corresponding trigger signal was also shown as green (contract) or red (relax) background color at a visual feedback display where the current force was shown as bar graph. Each 9th ISO was immediately followed by two BALs [(1) B40, (2) B20]. The resting period prior to the BALs was about 5 s. Together with the manual unlocking and fixation of the sledge, the duration of one fatigue set was about 1 min.

#### Subjects

Fifteen healthy male students of sport science completed the testing protocol correctly, which was checked using a usual documentation video (200 fps). The parameter identification results of six subjects had to be discarded because the final parameters values had converged toward solver boundary constraints. Consequently, the datasets of nine subjects remained ($27 \pm 3$ years, $183 \pm 8$ cm, BMI $22.4 \pm 1.6$ kg/m$^{2}$) and were analyzed throughout the fatigue task.

#### Data processing

The data processing was performed in Matlab^®^ 2016a. Force and position data were low-pass filtered at 15 Hz and 30 Hz for ISO and BAL contractions, respectively (zero-lag, butterworth, 4th $\mathscr {O}$) (Maffiuletti et al. 2016). The onset of force was automatically detected by a threshold which was set to the mean of the manually annotated silent period between the contractions plus 80 N (which is less than 5% of the max. force).

Some sEMG data showed frequency artifacts which were removed at 200 and 400 Hz using an adaptive notch filter. To reduce the overall standard deviation of the sEMG amplitude value, it was defined as the mean of two adjacent 0.05 s time windows which enclosed the maximum force per contraction (Merletti et al. 1990). At each period, the mean sEMG amplitude was calculated using wavelet analysis which represents a band pass filter between 6.9 and 395.5 Hz (Von Tscharner 2000) and was defined as the mean of the sum of the rectified wavelets (Borg 2010). For ISO, the sEMG muscle onsets and terminations were detected automatically by using the Teager–Kaiser energy operator and a threshold method (Kaiser 1990; Solnik et al. 2010). For BAL, the sEMG onsets were determined in the same way as for ISO, but due to the ongoing muscle contraction after push-off, the BAL terminations had to be set manually when force dropped to zero. Due to re-positioning and other little activities of the subjects during the breaks, the baseline signal had to be annotated by hand. Onsets which could be detected automatically were set by hand via a graphical interface.

### Mathematical model of the knee-extension task

The mathematical model and the parameter identification routine are discussed in detail in Penasso and Thaller (2017). This section provides a brief overview.

The model is formulated as an equation of motion that describes the time evolution of concentric and isometric knee-extension tasks as an ordinary differential equation which corresponds to the experimental setup (Fig. 1, Eq. 1). Each movement is initiated at the onset ($t_0$) of the time-dependent muscle activation dynamics (*A*(*t*)) which consider the progressing recruitment of fibers after onset (Eq. 2). *A*(*t*) considers the pre-activation level ($A_0$), the number of maximally available fibers ($n_{\rm max}$), and the rate constant (*U*) (Eq. 2). Once activated, the dynamics of the muscle–tendon complex (${\rm MTC}$) produce force ($f_{{\rm MTC}}$), where the actively force-generating element is the contractile element (CE). The CE is modeled as a contraction velocity ($\dot{l}_{{\rm CE}}$)-dependent force–velocity relation (FV) giving the CE force ($f_{\rm CE}(\dot{l}_{{\rm CE}})$) (Hill 1938) (Eq. 3). To consider length dependencies, its force is scaled by a parabolic force–length relation ($FL(l_{{\rm CE}})$), which is a function of the CE length ($l_{\rm CE}$) (Van Soest and Bobbert 1993) (Appendix A). It is assumed that the optimal length of the CE, representing a muscle fascicle, is 0.09 m which is reached at a knee-extension angle of 120$^\circ$ (Hoy et al. 1990). To be able to determine the value of the linear stiffness of the ${\rm SEE},$ it had to be set linear. The ${\rm SEE}$ depends on its length ($l_{{\rm SEE}}$) (Eq. 4) where the slack length is defined as the distance from the center of the patella to the tuberositas tibiae (cf. Sect. 2.1.1). The ratio of the external force of the model ($F_{\rm mod}$) and $f_{\rm MTC}$ is given by *G*(*X*),  which was published earlier (Sust et al. 1997b; Siebert et al. 2007; Wagner et al. 2006; Thaller et al. 2010) (Appendix B). Thus, $F_{{\rm mod}}(t)$ changes the position (*X*) of the point mass (*m*) during dynamic contractions. The displacement of *m* represents the movement of the sledge on a 10$^\circ$-inclined leg press,

1 $$\begin{aligned} F_{{\rm mod}}(t)&=m\ddot{X}+m\tilde{g} \nonumber \\&=G(X)\cdot \big (\underbrace{A(t)\cdot FL(l_{{\rm CE}})\cdot f_{{\rm CE}}(\dot{l}_{{\rm CE}})}_{f_{{\rm MTC}}}\big ), \end{aligned}$$

where $\tilde{g} = \sin (10\left[ ^{\circ }\right] ) \times 9.81 \ [\text {m}/ \text {s}^{2}]$. Thereby, *A*(*t*) is the solution of two coupled first-order differential equations, which consider time (*t*)-dependent excitation and recruitment of inactive muscle fibers.

2 $$\begin{aligned} A(t)= {\left\{ \begin{array}{ll} (A_0-n_{\rm max})\cdot \\ \exp \left( \frac{{\rm e}^{-t \cdot U} (-{\rm e}^{t \cdot U} (A_0+U (t \cdot U-1))+A_0-U)}{U}\right) \\ +n_{\rm max},&{} t \ge t_0,\\ A_0, &{} t<t_0, \end{array}\right. } \end{aligned}$$

which represents a normalized smooth sigmoid-like function ranging between the pre-activation level ($A_0$) and full activation ($n_{\rm max}=1$) with the slope controlled by the parameter of the rate constant of muscle activation (*U*). The FV is modeled according to Siebert et al. (2007) using Hill’s parameters *a*, *b*, and *c*

3 $$\begin{aligned} f_{{\rm CE}}(\dot{l}_{\rm CE})=\frac{c}{\underbrace{G(X)\cdot \dot{X}- \dot{l}_{{\rm SEE}}}_{\dot{l}_{\rm CE}}+b}-a, \end{aligned}$$

where *G*(*X*) is used to convert the external velocity ($\dot{X}$) to the velocity of the MTC ($v_{{\rm MTC}}=G(X)\cdot \dot{X}$), which is then used to calculate $\dot{l}_{{\rm CE}}$ by considering the velocity of the ${\rm SEE}$ ($\dot{l}_{{\rm SEE}} = v_{{\rm MTC}}-\dot{l}_{{\rm CE}}$). The ${\rm SEE}$ force ($f_{{\rm SEE}}$) corresponds to a linear spring

4 $$\begin{aligned} f_{{\rm SEE}}(\Delta l_{{\rm SEE}})=k_{{\rm SEE,lin}} \cdot \Delta l_{{\rm SEE}}, \end{aligned}$$

where the parameter $k_{{\rm SEE,lin}}$ represents the linear stiffness.

#### Parameter identification

To calculate the model parameters for all stages of fatigue, the parameter identification routine minimized the sum of squared errors between $\rm F_{z}$ of the force plate ($\rm F_{{\rm meas}}$) and the external force of the model ($F_{{\rm mod}}(t)$). It processes datasets containing isometric and dynamic ballistic contractions from different loads (ISO, B20, B40) at the same time, equally weighted. Since ISO and BAL contractions are differently affected by fatigue (Griffin et al. 2000; Morel et al. 2015), a contraction-type-dependent version of the identification routine was first tested using simulated data and then used to identify parameter sets of the fresh and fatigued states. This eight degrees of freedom least squares problem (including one $t_0$ for each of the three contractions per dataset) was solved numerically using the Matlab^®^ global search class together with the interior-point algorithm of the fmincon solver.

**Fig. 3:**
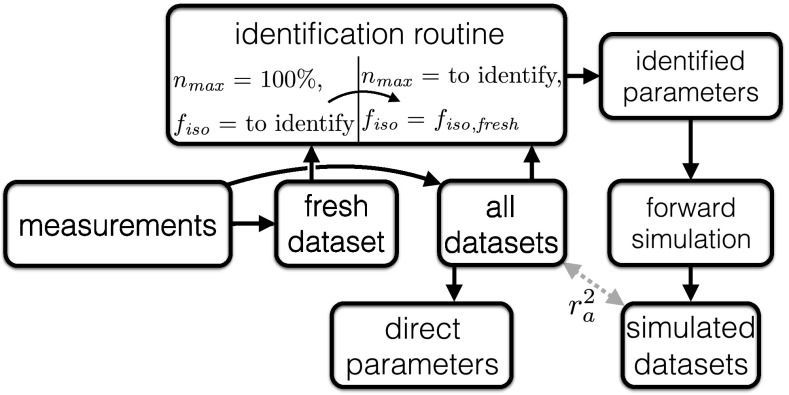
Flowchart illustrating the calculation of the direct parameters and the procedure of parameter identification

Initially, the parameters of the fresh state are determined (Fig. 3). The dataset consists of three contractions. Out of the first two B20, two B40, and three ISO contractions, each steepest and/or strongest contractions was selected automatically. It was assumed that the number of maximally available fibers corresponds to $n_{{\rm max}}$ = 100% and thus the parameter maximum isometric force ($f_{{\rm iso}}$) could be identified (Siebert et al. 2007). In the next step, model parameter values from all datasets are calculated. At each *n*th fatigued state, the 9th ISO contraction was processed together with the two subsequent dynamic MVCs (B40, B20). To assess if isometrically induced fatigue affects ISO and BAL tasks differently, $f_{{\rm iso}}$ of the fresh state ($f_{{\rm iso,fresh}}$) was set constant to enable the identification of model parameter values which are specific for BAL and ISO tasks (cf. “Optimized parameters”). Subsequently, each identified parameter set was used to simulate the corresponding movement and the adjusted correlation coefficient ($r^2_{\rm a}$) was calculated between the resulting simulated $F_{{\rm mod}}$ and measured data $\rm F_{{\rm meas}}$.

### Extracted parameters

Fatigue-related parameter trends of the Hill-type model muscle coupled with activation dynamics (optimized parameters) and values calculated from the measured force, position and sEMG data (direct values) were analyzed.

#### Optimized parameters

Contraction-type-dependent values were identified for the function activation dynamics of the muscle (Table 1). As fatigue causes motor unit activation decrease during maximum efforts (Bigland-Ritchie et al. 1979) while cellular inorganic phosphate reduces contractility (Stutzig et al. 2017), the maximum CE force generation capacity cannot be assigned to either $f_{{\rm iso}}$ or $n_{{\rm max}}$. $f_{\rm CE,max} = f_{{\rm iso}} \cdot n_{{\rm max}}$, $f_{{\rm iso}}$ was set to the identified value of $f_{{\rm iso,fresh}}$ for computational reasons and the parameters $n_{{\rm max,{\rm ISO}}}$, $n_{{\rm max,{\rm BAL}}}$, $U_{\rm ISO}$, $U_{\rm BAL}$ were identified at subsequent stages of the fatigue protocol (Fig. 3 and Eq. 2). Besides $f_{\rm iso}$, two further parameters were required to characterize the FV (Thaller and Wagner 2004). One parameter represents the maximum contraction velocity ($v_{\rm max}$), the other the maximum efficiency ($\eta _{\rm max}$) which is mathematically related to the curvature of the FV. Both were identified independently from contraction type. Furthermore, $k_{{\rm SEE,lin}}$ was identified to characterize the ${\rm SEE}$ behavior.

Parameters of $FL(l_{\rm CE})$ were not optimized because no substantial fatigue dependency is expected in normalized force–length relations (MacNaughton and MacIntosh 2006). The fatigue-related downward shift of the $FL(l_{\rm CE})$ is reflected in the parameters $n_{{\rm max,}{\rm ISO}}$ and $n_{{\rm max,}{\rm BAL}}$.

**Table 1:** Analyzed optimized parameters

Parameter	Equation		Technical meaning	Physiological meaning
$n_{{\rm max,{\rm ISO}}}$	*A*(*t*)	2	Scale CE force	Number of active fibers
$$n_{{\rm max,{\rm BAL}}}$$				
$U_{{\rm ISO}}$			Slope of *A*(*t*)	Max. fiber activation rate
$$U_{{\rm BAL}}$$				
$f_{\rm iso} = f_{{\rm iso,fresh}} \cdot n_{{\rm max}}$	$f_{{\rm CE}}(\dot{l}_{\rm CE})$	3	FV *y*-intercept	Max. isometric force
$\eta _{{\rm max}}$			FV curvature	Max. efficiency
$v_{{\rm max}}$			FV *x*-intercept	Max. contraction velocity
$k_{\rm SEE,lin}$	$f_{{\rm SEE}}(\Delta l_{{\rm SEE}})$	4	Spring constant	Elasticity of aponeurosis/tendon

*A*(*t*) activation dynamics, *CE* contractile element, *FV* force–velocity relation, *SEE* serial elastic element

Parameter conversions:

$$a=-\frac{f_{{\rm iso}} \left( \sqrt{\eta _{{\rm max}} }-1\right) ^2}{\eta _{{\rm max}} -2 \sqrt{\eta _{{\rm max}}}}$$, $$b=-\frac{\left( \sqrt{\eta _{{\rm max}} }-1\right) ^2 v_{{\rm max}}}{\eta _{{\rm max}} -2 \sqrt{\eta _{{\rm max}}}}$$, $$c=\frac{f_{{\rm iso}} \left( \sqrt{\eta _{{\rm max}} }-1\right) ^2 v_{{\rm max}}}{\left( \sqrt{\eta _{{\rm max}} }-2\right) ^2 \eta _{{\rm max}}}$$, $$p_{\rm max}=ab+c-2\sqrt{abc}$$, $$\eta _{\rm max}=p_{\rm max} /c$$

#### Direct values

Using filtered force (${\rm F}_{{\rm meas}}$) and position data ($\rm X_{{\rm meas}}$), the external maximum force $(\text {F}_{\text MAX}:= \max {\{\text{F}_{{\text {meas}}}}\})$, the maximum external power $(\text P_{\text{MAX}}:= \max {\{\text{F}_{{\text meas}}}\cdot \dot{\text{X}}_{{\rm meas}}\})$, and the maximum external velocity $(\text V_{\text MAX}:= \max { \{\dot{\text{X}}_{{\text {meas}}}\}})$ per contraction were obtained (Table 2). The rate of force development (RFD) was calculated at 50 ms after the detected force onset (Buckthorpe et al. 2014; Maffiuletti et al. 2016). The mean sEMG amplitudes (A) were calculated from the muscles of the right leg (e.g., $\rm A_{\rm VL}$ for m. vastus lateralis). The periods from the initiation and termination signal of the pacing trigger to the detected sEMG onset and termination events defined the reaction times. The duration of each contraction was defined as the duration between sEMG onset and termination.

**Table 2:** The analyzed direct parameters calculated at each contraction

Parameter	Description	Calculated as
Reaction time	Time between trigger signal and sEMG onset or termination	$t_{\rm sEMG\, onset}-t_{\rm trigger}$
Contraction duration	Time between sEMG onset and termination	$t_{\rm sEMG\, termination}-t_{\rm sEMG\, onset}$
$\rm F_{\rm MAX}$	External maximum force	$\max {\{\text{F}_{{\rm meas}}} \}$
$\rm P_{\rm MAX}$	The maximum external power	$\max \{ \text{F}_{\text{meas}} \cdot \dot{\text{X}}_{\text{meas}} \}$
$\rm V_{\rm MAX}$	The maximum external velocity	$\max \{ \dot{\text{X}}_{\text{meas}} \}$
RFD	Rate of force development at 50 ms after force-onset	$\rm F_{{\rm meas}}(50\,{\rm ms})/50\,{\rm ms}$
$\rm A_{\rm VL}$	sEMG amplitude m. vastus lateralis	cf. “Data processing”
$\rm A_{\rm VM}$	sEMG amplitude m. vastus medialis	cf. “Data processing”
$\rm A_{\rm RF}$	sEMG amplitude m. rectus femoris	cf. “Data processing”

### Statistics

Missing values at the time series were replaced by using the EM algorithm (Dempster et al. 1977). To analyze the time series of each parameter, the optimized parameters and direct values at every contraction number were first tested for the normality of the distribution by using the Kolmogorov–Smirnov test. At least once, the identified values of all subjects were not normally distributed; thus, the non-parametric Friedman test was used to assess if a time evolution changed significantly. To investigate if parameter trends were differently between contraction types, the slopes of the linear trends were calculated. These were normally distributed and thus were analyzed with a one-way ANOVA followed by Tukey HSD pairwise comparisons. The resulting *p* values are indicated as ***, **, *, ^t^ and reflect $p \le 0.001$, $p \le 0.01$, $p \le 0.05$, and $p \le 0.1$, respectively. The software package G*Power was used to assess the statistical power of each test (Faul et al. 2007), where a value of 0.8 was considered as sufficient statistical power. Effect sizes of the Friedman test were calculated using Kendall’s coefficient of concordance ($W_{\rm K}$) and ANOVA effect sizes were calculated using Hedge’s $g^*$. Effects where $0.2 \le W_{\rm K}{\text { or }}g^* < 0.5$ are classified as small ($_{\text {S}}$), $0.5 \le W_{\rm K}{\text { or }}g^* < 0.8$ as medium ($_{\text {M}}$), and $W_{\rm K}{\text { or }}g^* \ge 0.8$ as large ($_{\text {L}}$) and are reported only if the Friedman test or the Tukey HSD was significant.

## Results

The identification routine was successfully tested by re-identifying the input parameters of previously simulated datasets (relative errors $< 2.3 \times 10^{-4}$%). Thus, the identification of different values of $n_{{\rm max}}$ for ISO and BAL contractions is in accordance with previous model tests (Penasso and Thaller 2017). In 12.1% of all datasets, the identification routine converged toward bound constraints and thus these data had to be replaced with estimations based on the EM algorithm. The goodness of fit did not change in the course of fatigue (mean $r^2_{\rm a} = 0.99 \pm 0.009$, $p = 0.23$; mean normalized root-mean-squared error $=0.91\pm 0.02$, $p=0.15$) implying that the model was able to fit fresh and fatigued data in equal measure.

To test if the subjects rested sufficiently between the fresh contractions, $$\rm P_{\rm MAX}$$ of the fresh first BAL contraction was compared to the subsequent second contraction which was 99.7 ± 12.6% of the initial one. During the fatigue protocol, the subjects reacted 41.6% faster to the stop signal (initial 0.88 ± 0.19 s, final 0.37 ± 0.31 s) (0.2% replaced, $$^{***}_{\rm S}$$, power > 0.99). Thus, the duration of the ISOs changed from 3.6 ± 0.2 s to 3.1 ± 0.3 s (to 86%) (0.2% replaced, $$^{***}_{\rm M}$$, power > 0.99) and remained greater than the 3 s intended.

### Optimized parameters

**Fig. 4:**
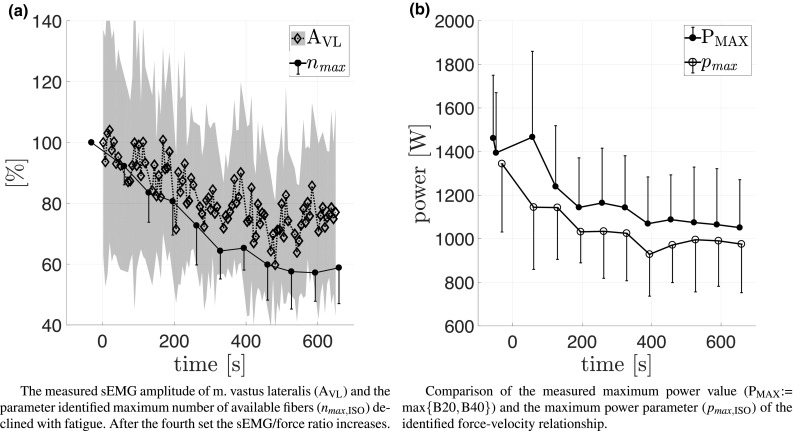
Comparison of related directly calculated values and identified parameters. As the first dynamic MVCs are preformed prior to the first isometric MVC, which defines ${\rm time}=0$, and as the identified parameter value timestamps represent the mean time per dataset, the timestamps of the fresh state and of the initital dynamic MVCs are shown at ${\rm times}<0$. Standard deviations are indicated as one-sided bars or shaded area

**Fig. 5:**
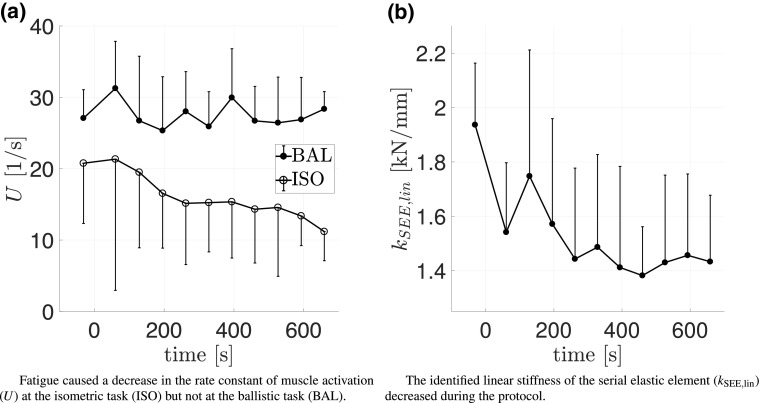
Effects of isometrically induced fatigue on identified parameter values. The first isometic MVC defines ${\rm time}=0$ and the optimized parameter value timestamps represent the mean time of the dataset. Standard deviations are indicated as one-sided bars

Using the identified value $f_{{\rm iso,fresh}} = 18{,}375\pm 3070$ N, the optimized parameters of fresh and fatigued states were identified (Table 3). The model parameters *U* and $n_{{\rm max}}$, describing the activation dynamics of the model muscle, were identified independently for ISO and BAL contractions. While the number of maximally available fibers $n_{{\rm max}}$ did not decrease significantly for the BAL tasks, $n_{{\rm max,}{\rm ISO}}$ decreased to $58.8\pm 11.9\%$ (Fig. 4a). Considering $f_{{\rm iso}} = f_{{\rm iso,fresh}} \cdot n_{{\rm max}}$, the behavior of $n_{{\rm max}}$ is also valid for $f_{{\rm iso}}$. Similar to $n_{{\rm max}}$, parameter changes of *U* were only detected for ISO ($U_{\rm ISO}$), which was reduced to $53.9\pm 22.1\%$ (Fig. 5a).

**Fig. 6:**
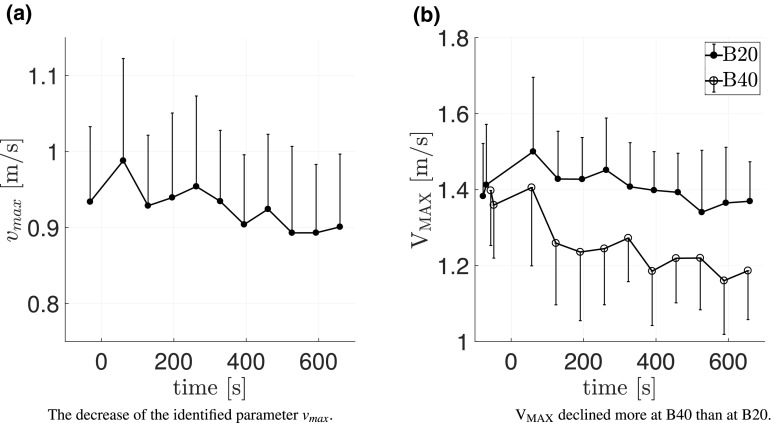
The effect of isometrically induced fatigue on **a** the identified parameter values of the maximum contraction velocity ($v_{\rm max}$) and **b** the measured maximum velocity (V$_{\rm MAX}$) at ballistic tasks with 40 kg (B40) and 20 kg (B20) extra load. The first isometic MVC defines ${\rm time}=0$ and the optimized parameter value timestamps represent the mean time of the dataset. Standard deviations are indicated as one-sided bars

For all contraction types, fatigue caused an increase of the FV curvature, which is mathematically related to the increase in $\eta _{\rm max}$ to $119.4\pm 19.7\%$. Since $\eta _{{\rm max}}=p_{{\rm max}}/c,$ the increase in $\eta _{\rm max}$ is also related to $p_{\rm max}$. Using $p_{\rm max}=ab+c-2\sqrt{abc}$ (Table 1) (Thaller and Wagner 2004), a decrease in $p_{\rm max}$ to $82.2\pm 13.5\%$ is seen (Fig. 4b). The third parameter characterizing the FV is $v_{\rm max},$ which was reduced to $96.5\pm 5\%$ (Fig. 6a).

The linear stiffness of the SEE, identified for ISO and BAL contractions together, and was reduced to $74\pm 7.5\%$ with fatigue (Fig. 5b).

**Table 3:** Mean values $\mu$ and standard deviations ($\sigma$) of optimized parameters at the fresh and final fatigued states

	ISO		BAL		$\eta _{{\rm max}}$	$v_{{\rm max}}$ (m/s)	$k_{\rm SEE,lin}$ (N/mm)
	$n_{{\rm max}}$	*U* (1/s)	$n_{{\rm max}}$	*U* (1/s)			
Fresh							
$\mu$	0.99	20.8	0.94	27.1	0.35	0.93	1936
($\sigma$)	(0.01)	(8.4)	(0.14)	(4)	(0.04)	(0.1)	(227)
Fatigued							
$\mu$	0.58	11.2	0.87	28.4	0.42	0.9	1432
($\sigma$)	(0.12)	(4.1)	(0.15)	(2.4)	(0.05)	(0.1)	(245)
	$$_{\text {L}}$$ $$^{***}_{\dagger \dagger \dagger }$$	$$_{\text {L}}$$ $$^{*}_{\dagger }$$	–	–	$$_{\text {L}}$$ $$^{**}_{\dagger \dagger }$$	$$_{\text {L}}$$ $$^{***}_{\dagger \dagger }$$	$$_{\text {L}}$$ $$^{**}_{\dagger \dagger }$$

At the isometric task (ISO), the number of active motor fibers ($n_{{\rm max}}$) and the activation rate constant (*U*) declined, whereas isometrically induced fatigue did not affect these parameters at ballistic tasks (BAL). Reductions in the parameter values of max. efficiency ($\eta _{{\rm max}}$), max. contraction velocity ($v_{{\rm max}}$), and linear stiffness of the serial elastic element ($k_{\rm SEE,lin}$) were found. The parameter description is given in Table 1

Statistical significance, power, and effect sizes are shown in the last row

***$p \le 0.001$, **$p \le 0.01$, *$p \le 0.05$; $_{\dagger \dagger \dagger }$ power $> 0.99$, $_{\dagger \dagger }$ power $\ge 0.9$, $_{\dagger }$ power $\ge 0.8$; $_{\text {S}}$ small effect, $_{\text {M}}$ medium effect, $_{\text {L}}$ large effect

### Direct values

Using the maximum value out of the first three ISO contractions and the maximum value of the first two contractions of B40 and B20, the fresh states (100%) were calculated. The majority of the directly measured values calculated from force and position data as well as the sEMG amplitude declined with fatigue (Table 4). The slopes of the linear parameter trends calculated from force and position data indicate that the isometrically induced fatigue affects faster contractions less than slower ones (Table 5).

**Fig. 7:**
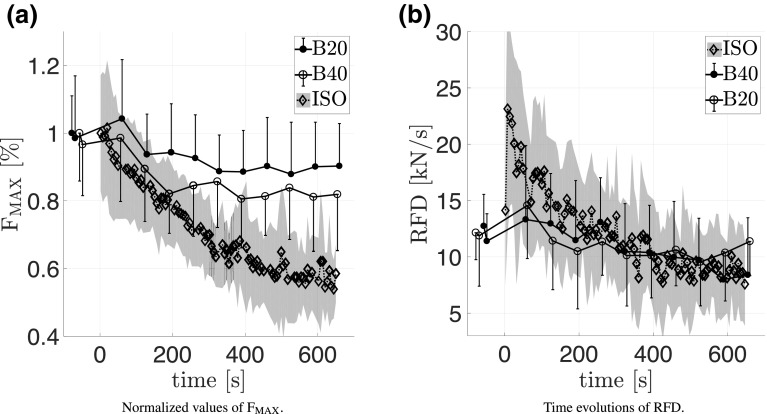
The directly measured values of the maximum force per contraction ($\rm F_{\rm MAX}$) and of the rate of force development (RFD) decreased with fatigue. The decline was greater at the isometric task (ISO) than at both dynamic ballistic tasks (B40 and B20). The first isometic MVC defines ${\rm time}=0$. Standard deviations are indicated as one-sided bars or shaded areas

Figure 7a shows that $\rm F_{\rm MAX}$ dropped to $53.2\pm 13$, $80.1\pm 14.8$ and $84.8\pm 12.3\%$ at ISO, B40 and B20 contractions, respectively. Thereby, the values of the slopes of the linear trends showed greater reductions in the ISO task than at BAL. The reduction of $\rm P_{\rm MAX}$ at B40 and B20 to $68.6 \pm 21.2$ and $82.4\pm 15.6\%$ is shown in Fig. 4b. The decline at the slower and higher force requiring B40 task was greater than at the B20 task. Differences in the trends of $\rm V_{\rm max}$ at B40 and B20 contractions are illustrated in Fig. 6b, which declined to $84.3\pm 11.4$ and $93.8\pm 7.5\%$. RFD was reduced to $25\pm 13.4$, $57.4\pm 38.6$ and $65.6\pm 27.1\%$ for ISO, B40, and B20, respectively, where ISO showed stronger fatigue effects than BAL (Fig. 7b).

Most of the sEMG amplitudes of the knee-extensor muscles decreased with fatigue. Figure 4a shows the reduction of $\rm A_{\rm VM}$ at ISO contractions to $63.5 \pm 19.9\%$. At B40, $\rm A_{\rm VM}$ was reduced to $72.4 \pm 25.3\%$. $\rm A_{\rm VL}$ decreased to $59.8\pm 15\%$ for ISO and a tendency was found for B40 $69.7\pm 23.3\%$. At ISO, $\rm A_{\rm RF}$ showed a reduction to $53.4 \pm 16.2\%,$ and at B40, a tendential reduction to $70.4 \pm 37.9\%$ was found. The slopes of the linear regressions of the sEMG amplitudes $\rm A_{\rm VM}$, $\rm A_{\rm VL}$, and $\rm A_{\rm RF}$ were not different for the ISO, B40, and B20 contractions (Table 5).

**Table 4:** Mean values $\mu$ and standard deviations ($\sigma$) of fresh and fatigued direct values

	Fresh			Fatigued		
	ISO	B40	B20	ISO	B40	B20
$$\rm F_{\rm MAX}$$ (N)						
$\mu$	3676	1258.5	1073.9	1954.9	1007.8	911.14
($\sigma$)	(616.5)	(177.23)	(147.02)	(435.26)	(195.79)	(144.15)
$$\rm P_{\rm MAX}$$ (W)						
$\mu$	–	1447	1205	–	992.6	992.7
($\sigma$)	(–)	(316.2)	(261.2)	(–)	(294)	(228.9)
$$\rm V_{\rm MAX}$$ (m/s)						
$\mu$	–	1.4	1.4	–	1.2	1.4
($\sigma$)	(–)	(0.14)	(0.12)	(–)	(0.15)	(0.14)
RFD (kN/s)						
$\mu$	27	13.2	13.4	6.76	7.59	8.8
($\sigma$)	(8.88)	(2.65)	(2.93)	(3.53)	(4.75)	(3.2)
$${\rm A}_{\rm VM} \ (\mu \text {V})$$						
$\mu$	1.2	1.3	1.3	0.75	0.93	1.1
($\sigma$)	(0.34)	(0.36)	(0.4)	(0.3)	(0.41)	(0.4)
$$\rm A_{\rm VL} \ (\mu \text {V})$$						
$\mu$	0.76	0.79	0.77	0.46	0.55	0.59
($\sigma$)	(0.3)	(0.12)	(0.21)	(0.18)	(0.24)	(0.19)
$$\rm A_{\rm RF} \ (\mu \text {V})$$						
$\mu$	0.48	0.55	0.57	0.26	0.39	0.48
($\sigma$)	(0.25)	(0.14)	(0.17)	(0.15)	(0.28)	(0.24)

For isometric tasks (ISO), the maximum value of the first three contractions and for the ballistic contractions with 40 kg (B40) and 20 kg (B20) extra load, the maximum of the first two contractions per subject is compared to the value calculated from the last contraction. The parameter description is given in Table 2

Significance and effect size of time series analysis and differences between linear slopes for ISO, B40, and B20 tasks of max. force per contraction ($\rm F_{\rm MAX}$), max.

power ($\rm P_{\rm MAX}$), max. velocity ($\rm V_{\rm MAX}$), rate of force development (RFD), as well as surface electromyographic amplitudes of m. vastus medialis ($\rm A_{\rm VM}$), m. vastus lateralis ($\rm A_{\rm VL}$), and m. rectus femoris ($\rm A_{\rm RF}$) are shown in Table 5

**Table 5:** Mean values $\mu$ and standard deviations ($\sigma$) of the slopes of the linear trends of directly measured values

	ISO	B40	B20	*ANOVA*
$$\rm F_{\rm MAX}$$ (N/s)				
$$\mu$$	$$_{\text {L}}$$− 2.1$$^{***}_{\dagger \dagger \dagger }$$	$$_{\text {L}}$$− 0.29$$^{***}_{\dagger \dagger }$$	$$_{\text {L}}$$− 0.17$$^{**}_{\dagger \dagger }$$	^***^
($$\sigma$$)	(0.82)	(0.2)	(0.13)	$$_{\text {L}}$$^***^$$^{\text {ISO}}_{\text {B}40}$$, $$_{\text {L}}$$^***^$$^{\text {ISO}}_{\text {B}20}$$
$$\rm P_{\rm MAX}$$ (W/s)				
$$\mu$$	–	$$_{\text {L}}$$− 0.57$$^{***}_{\dagger \dagger }$$	$$_{\text {L}}$$− 0.27$$^{**}_{\dagger \dagger }$$	^*^
($$\sigma$$)	–	(0.3)	(0.22)	$$_{\text {L}}$$ ^*^ $$^{\text {B}40}_{\text {B}20}$$
$\rm V_{\rm MAX}$ (m/s$^2$)				
$$\mu$$	–	$$_{\text {L}}$$ $$-2.8 \times 10^{-4}$$ $$^{***}_{\dagger \dagger }$$	$$_{\text {L}}$$ $$-1 \times 10^{-4}$$ $$^{*}_{\dagger \dagger }$$	^*^
($$\sigma$$)	–	$$(1.5 \times 10^{-4}$$)	($$1.3 \times 10^{-4}$$)	$$_{\text {L}}$$ ^*^ $$^{\text {B}40}_{\text {B}20}$$
RFD (N/s$^2$)				
$$\mu$$	$$_{\text {L}}$$− 15.7$$^{***}_{\dagger \dagger \dagger }$$	$$_{\text {L}}$$− 6.7$$^{**}_{\dagger \dagger }$$	$$_{\text {L}}$$− 3.7$$^{\text {t}}_{\dagger }$$	^***^
($$\sigma$$)	(6.5)	(4.6)	(5.4)	$$_{\text {L}}$$^**^$$^{\text {ISO}}_{\text {B}40}$$, $$_{\text {L}}$$^***^$$^{\text {ISO}}_{\text {B}20}$$
$$\rm A_{\rm VM} \ ({\mu \text {V}}/{\text {s}})$$				
$$\mu$$	$$_{\text {L}}$$ $$-4 \times 10^{-4}$$ $$^{***}_{\dagger \dagger \dagger }$$	$$_{\text {L}}$$ $$-4 \times 10^{-4}$$ $$^{*}_{\dagger \dagger }$$	$$-2.1 \times 10^{-4}$$	–
($\sigma$)	($2.2 \times 10^{-4}$)	($4.3 \times 10^{-4}$)	($2.1 \times 10^{-4}$)	–
$$\rm A_{\rm VL} \ ({\mu \text {V}}/{\text {s}})$$				
$$\mu$$	$$_{\text {L}}$$ $$-2.5 \times 10^{-4}$$ $$^{***}_{\dagger \dagger \dagger }$$	$$_{\text {L}}$$ $$-1.6 \times 10^{-4}$$ $$^{{\rm t}}_{\dagger }$$	$$-1.1 \times 10^{-4}$$	–
($\sigma$)	($2 \times 10^{-4}$)	($1.9 \times 10^{-4}$)	($1.5 \times 10^{-4}$)	–
$$\rm A_{\rm RF} \ ({\mu \text {V}}/{\text {s}})$$				
$$\mu$$	$$_{\text {L}}$$ $$-2.7 \times 10^{-4}$$ $$^{***}_{\dagger \dagger \dagger }$$	$$_{\text {L}}$$ $$-1.7 \times 10^{-4}$$ $$^{\text {t}}_{\dagger }$$	$$-8 \times 10^{-5}$$	–
($\sigma$)	($2.2 \times 10^{-4}$)	($2.4 \times 10^{-4}$)	($1.9 \times 10^{-4}$)	–

Most values declined with fatigue, and different effects of isometrically induced fatigue between isometric tasks (ISO) and ballistic tasks with 40 kg (B40) and 20 kg (B20) extra load are seen in max. force per contraction ($\rm F_{\rm MAX}$), max. power ($\rm P_{\rm MAX}$), max. velocity ($\rm V_{\rm MAX}$), and rate of force development (RFD). Surface electromyographic amplitudes of m. vastus medialis ($\rm A_{\rm VM}$), m. vastus lateralis ($\rm A_{\rm VL}$), and m. rectus femoris ($\rm A_{\rm RF}$) declined only at ISO and B40 tasks

Statistical significance, power, and effect sizes of the time series analysis are indicated as super- and subscript signs. The one-way ANOVA significance level and effect sizes $g^*$ are declared in the right column where the pairwise comparisons are indicated

***$p \le 0.001$, **$p \le 0.01$, *$p \le 0.05$, ^t^$p \le 0.1$; $_{\dagger \dagger \dagger }$ power $> 0.99$, $_{\dagger \dagger }$ power $\ge 0.9$, $_{\dagger }$ power $\ge 0.8$; $_{\text {S}}$ small effect, $_{\text {M}}$ medium effect, $_{\text {L}}$ large effect

### Comparison between optimized parameters and direct values

Our model-based analysis allowed the comparison between optimized parameters and values calculated directly from measured data. Using isometric MVCs at assumed optimal CE lengths, only *G*(*X*) determines the discrepancy between the maximum force-generating capacity of the CE ($f_{{\rm iso}}$ and/or $n_{{\rm max}}$) and $\rm F_{\rm MAX}$. Thus, their normalized values should fit (Figs. 4a, 7a).

The values of $\rm P_{\rm MAX}$ and the FV parameter $p_{\rm max}$ can be directly compared (Fig. 4(b)). However, $\rm P_{\rm MAX}$ must be greater than $p_{\rm max}$ at ballistic contractions, because energy is initially stored in the SEE and released later (Komi 1984).

The difference in the values of the measured maximum velocity per contraction ($\rm V_{\rm MAX}$) and the FV parameter maximum velocity ($v_{\rm max}$) arises from the *G*(*X*) as well as from the interplay of CE and SEE. Thereby, $\rm V_{\rm MAX}$ represents the transferred sum of the velocity of CE and SEE, whereas $v_{\rm max}$ represents the maximum possible velocity of the CE and even may not be reached during the movement (Fig. 6).

Considering that fiber activation thresholds increase with fatigue (Grabowski et al. 1972) and that calcium sensitivity decreases (Westerblad and Allen 1991), the normalized trends of the values of the rectified and smoothed sEMG and the value of $f_{{\rm iso}}$ and/or $n_{{\rm max}}$ are expected to separate with fatigue (Fig. 4a). Thus, sEMG amplitudes of fatigued contractions cannot be easily converted to the active state of activation dynamics, which becomes even more difficult for dynamic contractions (Disselhorst-Klug et al. 2009). These effects also apply to RFD which also highly depends on the interval used for calculation. Thus, neither RFD nor slope values calculated from sEMG amplitude can be compared to the slope parameter of the activation dynamics *U* (Fig. 5a).

## Discussion

Especially in modeling dynamic movements, the geometric configuration of the legs (Bobbert 2012), the dynamics of contractile and elastic elements (Penasso and Thaller 2017), as well as the state of muscular activation (Siebert et al. 2007) determine the resulting movement (Bernstein 1967). For example, the external maximum power ($\rm P_{\rm MAX}$) must be greater than the parameter maximum power ($p_{\rm max}$) of the CE, because the externally measured values represent the combined dynamics of the CE and SEE. Thereby, a parameter is a property of a structure, e.g., a muscle, which does not depend on the measurement condition or any other variable. Thus, movement-dependent values, e.g., RFD which is different for ISO and BAL contractions (Table 4), should not be used as model parameters.

Parameters reflecting the geometric relations can be measured directly and may be used within geometric functions to calculate internal forces. As the geometric function incorporates the knee as a hinge joint with constant lever arm, a more detailed description of the knee, e.g., Van Eijden et al. (1986), would change the identified values. The resulting parameter trends would be shifted. However, their trends would follow the same patterns because the range of motion was constant. Using parameter optimization methods, these internal forces can be reconstructed by a model of force generation, e.g., a Hill-type muscle model. These are characterized by parameter values that, within the range of validity of the model, do not depend on the movement modality. The curvature of the muscle FV is such a parameter and can be determined using the modeling approach. It is related to the individual fiber-type distribution (Sust et al. 1997b; MacIntosh et al. 1993), which cannot be extracted from simple measurements without an underlying model.

To determine the internal parameter values within physiological limits, a constrained parameter identification routine was used to optimize the parameters to fit the measured knee-extension data of fresh and continuously fatigued subjects. This allowed us to establish fatigue-related model parameter trends of *A*(*t*), FV, and SEE, which also revealed task-specific effects of isometrically induced fatigue regarding ISO and BAL tasks.

The analysis of the linear trends showed that most of the externally measured values declined more for ISO compared to the subsequent BAL tasks. In general, isometrically induced fatigue effected slow high-force tasks more than fast low-force tasks (Tables 3, 4, 5). Similar results were already reported by Morel et al. (2015), who showed that the magnitude of muscle activation was more affected at ISO tasks than at fast dynamic tasks, which seemed to be mainly affected by peripheral fatigue factors. As the parameters of *A*(*t*) also showed such a behavior, our findings could be explained by similar mechanisms. Thereby, the rate constant of muscle activation (*U*) and the maximum capacity of force generation of the CE (related to the number of maximally available fibers $n_{\rm max}$) declined at ISO only (Fig. 5a). Thus, it is likely that the BAL tasks were mainly affected by fatigue-related changes in the peripheral system (e.g., excitation–contraction coupling, cross-bridge dynamics), whereas the ISO tasks had been influenced by a combination of central and peripheral factors.

At very low load dynamic movements performed with maximum effort, it is possible that higher-threshold motor units are not recruited (Milner-Brown et al. 1973). Furthermore, if a dynamic movement lasts less than the time needed for full activation, only a part of the whole amount of motor units is activated. Thus, it is likely that several higher-threshold motor units are not recruited.

The fatigue effects on the CE resulted in an increase in the FV maximum efficiency ($\eta _{\rm max}$), while the maximum contraction velocity ($v_{\rm max}$) was reduced. As the weaker lower-threshold motor units (slow-type I) muscle fibers also have a higher value of $\eta _{\rm max}$ (MacIntosh et al. 1993; Sust et al. 1997b), this shift can be explained by the fatigue-related de-activation of high-threshold motor units (fast-type II) (Fitts 2008; Johnson et al. 1973; Westad et al. 2003). Another explanation is the single fiber force reduction due to inorganic phosphate accumulation (Stutzig et al. 2017).

Together with the active elements, the passive SEE became less stiff ($74\pm 7.5\%$, Fig. 5b). A similar increase in the compliance of the SEE was reported by an ultrasound study of Kubo et al. (2001), who showed that the m. vastus lateralis tendon and aponeurosis stiffness decreased to 72% after 50 sets consisting of 3 s MVC and 3 s relaxation. Furthermore, their results indicated that the increase in compliance was mainly related to the duration of the contraction and not to the functioning of the muscle.

### Limitations

Our knee-extensor muscle model represents the combined function of all muscles involved and thus does not account for synergistic and/or antagonistic force sharing (Stutzig and Siebert 2015; Herzog and Leonard 1991). Subject- and sport-related adaptations in force–length relations were not considered (Herzog et al. 1991), thus, such effects are absorbed by the optimized parameters, but should be negligible compared to the induced fatigue effects. As the model represents a more general mechanical level, cellular mechanisms (Allen et al. 2008; Fitts 1994; MacIntosh et al. 2012; Place et al. 2010; Rzanny et al. 2016), as well as effects of non-constant recruitment thresholds of muscle fiber types (Bigland-Ritchie and Woods 1984), were not modeled. Central and peripheral fatigue effects were considered in the data analysis, but an underlying model that could explain the task dependency of fatigue was not included.

Extracting model parameter values from maximum voluntary contractions is always subject to uncertainties related to the achieved magnitude of effort. For example, the first ISO was often performed more cautiously than the subsequent ones, where one of the second to fourth contractions showed the highest force and/or rate of force development (Fig. 7a). Previous works had shown that the model is able to simulate the knee-extension movement if the model requirements are met (Sust et al. 1997b; Siebert et al. 2008). By comparing a similar modeling approach and the electroencephalography mean spectra theta amplitude Sust et al. (1997a) showed that poor force fits (33% of their data) seemed to coincide with sub-maximal contractions.

As the solution space of our parameter identification routine is relatively flat, only one contraction, performed submaximally or with non-constant effort, is able to create false attractors for the solver. This would lead to unstable and unphysiological results that are excluded using bound constraints. Especially when fatigue sets in, the physical performance highly depends on the individual perception of fatigue (Noakes et al. 2005). Subjects might get used to repeated constant encouragement with the repetitions and pain increases. Thus, some parameter identification problems may be related to short-term motivational problems impairing the subjects’ ability to reliably perform MVCs at fatigued states (MacIntosh et al. 1993). As the parameter identification was again successful after it had failed at a level of fatigue, it is reasonable to explain the 12.1% replaced datasets with unsuccessful MVC attempts. The amount of 40% excluded subjects seems not drastically higher than the reported 33% value of Sust et al. (1997a) if one considers that the strict protocol did not allow second MVC attempts and MVCs had to be performed under more difficult conditions. Thus, only data of subjects who, despite fatigue sensations, were able to very reliably perform MVCs could be included. Consequently, the reported values characterize only highly motivated active young healthy males. Reducing the amount of data loss by specifically training the subjects (Maffiuletti et al. 2016) and by using motivating interventions such as rewards would clearly improve the approach. However, it is known that the fresh parameter values vary individually and between different populations (Thaller and Wagner 2004; Wagner et al. 2006). Thus, in dependence of the initial state, the magnitude of the fatigue response should vary individually and between populations. The general behavior, however, should be the same.

History effects of contractions are known since the early 1940s (Abbott and Aubert 1952). Force depression, denoting reduced isometric force, occurs if an isometric contraction is performed after a shortening contraction against resistance (Siebert et al. 2008), but disappears if shortening continues (Till et al. 2014). Force enhancement describes the opposite, where following a lengthening contraction the isometric force is increased (Herzog 1998). These phenomena can be explained by calcium-controlled mechanisms where titin binds to the actin filament (Herzog 2014; Rode et al. 2009). Furthermore, calcium sensitivity of the myosin light chain is increased by preceding contractions, thus improving muscular performance (postactivation potentiation) (Sale 2002). As our protocol included isometric and ballistic contractions prior to further contractions, the effects of postactivation potentiation, fatigue, and to some extend also force depression overlap and might compensate and/or amplify each other. Consequently, our identified parameter values are influenced by these basic mechanisms. Only more detailed cross-bridge models would be able to consider such and further dependencies, e.g., length-dependent activation, but require more parameters. This, however, facilitates that the model output is only barely sensitive to some parameters (Rockenfeller et al. 2015) and as a consequence parameter identification is not applicable to these hardly measurable parameters.

To assess the effect of the rapid central and peripheral recovery within the first 2 min (Froyd et al. 2013; Gruet et al. 2014), we tested if the short recovery periods prior to ISO tasks ($\sim$ 3 s) and to the B40 and B20 tasks ($\sim$ 5 s) had substantial influence on the contractile properties of the muscles. A Wilcoxon signed-rank test showed that neither RFD nor $\rm F_{\rm MAX}$ of the ninth ISO per set was different to the first ISO of the subsequent set. Thus, the subjects’ muscles seem not to have recovered significantly during the ballistic contractions. However, it is important to note that the subjects always looked forward to the BAL contractions because these where less painful than the ISO contractions in the fatigue conditions. This motivational boost in favor of BAL certainly contributed to the observed differences between ISO vs. BAL, but less to the differences between B40 vs. B20 (cf. Tables 4, 5). Furthermore, also the strict sequence of contractions (ISO–B40–B20) might have influenced the results. Despite these limitations, our results reflect how a Hill-type muscle model with activation dynamics should typically adapt to fatigue. We have demonstrated that muscle activation is crucially related to task dependency and that CE as well as SEE parameters change with fatigue.

Even though our parameter identification approach is limited to contractions performed with maximum effort, widespread simulation models of fresh movements, which were also validated in the submaximal case (e.g., OpenSim), principally rely on parameter values similar to ours. In contrast to models using parameter values of fresh muscles, for modeling fatigued movements the recovery process must also be taken into account. In long-duration submaximal movements, inactive fibers recover or fresh ones are activated additionally, thus changing the fatigue state of the whole muscle. However, in short submaximal movements, these effects may be neglected and therefore the identified muscle parameters may be used for simulations.

### Conclusions

Using a modeling and parameter identification approach, it was possible to determine fatigue-related trends of Hill-type muscle model parameters and activation dynamics from simple measurements. The use of the mathematical muscle model allowed us to study the effects of fatigue at a macroscopic level. Therefore, our results can be used within other Hill-type muscle models for short-duration movements.

According to our results, models intended for simulating movements of fatigued subjects should consider fatigue-related parameter dependencies in the sub-models of contractile element, and serial elastic element. Additionally, the muscle activation dynamics should account for contraction-type dependencies if simulations of mixed task modalities are intended. To reveal the underlying neurological control of the task dependency, further studies would be beneficial for creating detailed fatigue models.

## Appendix A: Force–length relationship

The force–length relationship is taken from Van Soest and Bobbert (1993) and is defined within the lengths $(1-{\rm WIDTH}) \cdot l_{M,{\rm OPT}}$ and $(1+{\rm WIDTH}) \cdot l_{M,{\rm OPT}}$:

5 $$\begin{aligned} {FL}(l_{\rm CE})&= \zeta \cdot \left( \dfrac{l_{\rm CE}}{l_{{\rm CE},{\rm OPT}}}\right) ^2-2 \zeta \cdot \left( \dfrac{l_{\rm CE}}{l_{{\rm CE},{\rm OPT}}}\right) + \zeta +1, \nonumber \\&\quad {\rm using} \quad \zeta = -\dfrac{1}{{\rm WIDTH}^{2}}, \end{aligned}$$

where $l_{{\rm CE},{\rm OPT}}$ is the optimum length of the CE and ${\rm WIDTH}=0.56$.

## Appendix B: Geometrical relations

The ratio of $F_{\rm mod}$ and $f_{\rm MTC}$ is given by

6 $$\begin{aligned} G(X)=\dfrac{r \cdot \sin (\beta )\cdot X}{l_{\rm thigh} \cdot l_{\rm shank} \cdot \sin (\alpha )} \end{aligned}$$

(Sust et al. 1997b; Siebert et al. 2007; Wagner et al. 2006; Thaller et al. 2010). Thereby, it is assumed that $l_{\rm thigh}$, the length of the model muscle–tendon complex, is equal to the length of the thigh, $l_{\rm shank}$ is the length of the shank, *r* is the moment arm of the knee, $\alpha$ is the knee angle calculated via the cosine rule, and $\beta (\alpha )$ is calculated via numerical minimization (Fig. 1).

## Abbreviations

A: Amplitude of sEMG
*A*(*t*): Activation dynamics
B20: BAL with $33.5 + 20$ kg load
B40: BAL with $33.5 + 40$ kg load
BAL: Ballistic contraction task
CE: Contractile element
*FL*: Force–length relation
FV: Force–velocity relation
ISO: Isometric contraction task
MTC: Muscle tendon complex
MVC: Maximum voluntary contraction
$r^2_{\rm a}$: Adjusted coefficient of correlation
RF: M. rectus femoris
RFD: Rate of force development
SEE: Serial elastic element
sEMG: Surface electromyography
SR: Sampling rate
VL: M. vastus lateralis
VM: M. vastus medialis

## References

[CR1] Abbott BC, Aubert XM (1952). The force exerted by active striated muscle during and after change of length. J Physiol.

[CR2] Allen DG, Lamb GD, Westerblad HK (2008). Skeletal muscle fatigue: cellular mechanisms. Physiol Rev.

[CR3] Babault N, Desbrosses K, Fabre MS, Michaut A, Pousson M (2006). Neuromuscular fatigue development during maximal concentric and isometric knee extensions. J Appl Physiol (1985).

[CR4] Bernstein NA (1967). The co-ordination and regulation of movements.

[CR5] Bigland-Ritchie B, Woods JJ (1984). Changes in muscle contractile properties and neural control during human muscular fatigue. Muscle Nerve.

[CR6] Bigland-Ritchie B, Jones DA, Hosking GP, Edwards RH (1978). Central and peripheral fatigue in sustained maximum voluntary contractions of human quadriceps muscle. Clin Sci Mol Med.

[CR7] Bigland-Ritchie B, Jones DA, Woods JJ (1979). Excitation frequency and muscle fatigue: electrical responses during human voluntary and stimulated contractions. Exp Neurol.

[CR8] Bobbert MF (2012). Why is the force–velocity relationship in leg press tasks quasi-linear rather than hyperbolic?. J Appl Physiol (1985).

[CR9] Borg F (2010) Filter banks and the “Intensity Analysis” of EMG. arXiv:1005.0696, arXiv:1005.0696

[CR10] Buckthorpe M, Pain MTG, Folland JP (2014). Central fatigue contributes to the greater reductions in explosive than maximal strength with high-intensity fatigue. Exp Physiol.

[CR11] Callahan DM, Umberger BR, Kent JA (2016). Mechanisms of in vivo muscle fatigue in humans: investigating age-related fatigue resistance with a computational model. J Physiol.

[CR12] Dempster AP, Laird NM, Rubin DB (1977) Maximum likelihood from incomplete data via the EM algorithm. J R Stat Soc Series B Stat Methodol 39(1):1–38. http://www.jstor.org/stable/2984875

[CR13] Disselhorst-Klug C, Schmitz-Rode T, Rau G (2009). Surface electromyography and muscle force: Limits in sEMG-force relationship and new approaches for applications. Clin Biomech.

[CR14] Edman KA, Mattiazzi AR (1981). Effects of fatigue and altered pH on isometric force and velocity of shortening at zero load in frog muscle fibres. J Muscle Res Cell Motil.

[CR15] Enoka RM, Duchateau J (2016). Translating fatigue to human performance. Med Sci Sports Exerc.

[CR16] Enoka RM, Stuart DG (1992). Neurobiology of muscle fatigue. J Appl Physiol (1985).

[CR17] Faul F, Erdfelder E, Lang AG, Buchner A (2007). G*Power 3: a flexible statistical power analysis program for the social, behavioral, and biomedical sciences. Behav Res Methods.

[CR18] Fitts RH (1994). Cellular mechanisms of muscle fatigue. Physiol Rev.

[CR19] Fitts RH (2008). The cross-bridge cycle and skeletal muscle fatigue. J Appl Physiol (1985).

[CR20] Froyd C, Millet GY, Noakes TD (2013). The development of peripheral fatigue and short-term recovery during self-paced high-intensity exercise. J Physiol.

[CR21] Gandevia SC (2001). Spinal and supraspinal factors in human muscle fatigue. Physiol Rev.

[CR22] Gandevia SC, Herbert RD, Leeper JB (1998). Voluntary activation of human elbow flexor muscles during maximal concentric contractions. J Physiol.

[CR23] Grabowski W, Lobsiger EA, Lüttgau HC (1972). The effect of repetitive stimulation at low frequencies upon the electrical and mechanical activity of single muscle fibres. Pflugers Arch.

[CR24] Griffin L, Ivanova T, Garland SJ (2000). Role of limb movement in the modulation of motor unit discharge rate during fatiguing contractions. Exp Brain Res.

[CR25] Gruet M, Temesi J, Rupp T, Levy P, Verges S, Millet GY (2014). Dynamics of corticospinal changes during and after high-intensity quadriceps exercise. Exp Physiol.

[CR26] Harwood B, Davidson AW, Rice CL (2011). Motor unit discharge rates of the anconeus muscle during high-velocity elbow extensions. Exp Brain Res.

[CR27] Hawkins D, Hull ML (1993). Muscle force as affected by fatigue: mathematical model and experimental verification. J Biomech.

[CR28] Hermens HJ, Freriks B, Disselhorst-Klug C, Rau G (2000). Development of recommendations for SEMG sensors and sensor placement procedures. Electromyogr Kinesiol.

[CR29] Herzog W (1998). History dependence of force production in skeletal muscle: a proposal for mechanisms. J Electromyogr Kinesiol.

[CR30] Herzog W (2014). Mechanisms of enhanced force production in lengthening (eccentric) muscle contractions. J Appl Physiol (1985).

[CR31] Herzog W, Leonard TR (1991). Validation of optimization models that estimate the forces exerted by synergistic muscles. J Biomech.

[CR32] Herzog W, Guimaraes AC, Anton MG, Carter-Erdman KA (1991) Moment-length relations of rectus femoris muscles of speed skaters/cyclists and runners. Med Sci Sports Exerc 23(11):1289–1296. https://www.ncbi.nlm.nih.gov/pubmed/1766346 1766346

[CR33] Hill AV (1938). The heat of shortening and the dynamic constants of muscle. Proc R Soc Lond B Biol Sci.

[CR34] Hilty L (2011) Cerebral processes mediating muscle fatigue in healthy humans. Ph.D. thesis, ETH Zürich. 10.3929/ethz-a-006680212

[CR35] Hoy MG, Zajac FE, Gordon ME (1990). A musculoskeletal model of the human lower extremity: the effect of muscle, tendon, and moment arm on the moment-angle relationship of musculotendon actuators at the hip, knee, and ankle. J Biomech.

[CR36] Johnson MA, Polgar J, Weightman D, Appleton D (1973). Data on the distribution of fibre types in thirty-six human muscles. An autopsy study. J Neurol Sci.

[CR37] Jones DA, De Ruiter CJ, De Haan A (2006). Change in contractile properties of human muscle in relationship to the loss of power and slowing of relaxation seen with fatigue. J Physiol.

[CR38] Kaiser JF (1990) On a simple algorithm to calculate the ‘energy’ of a signal. In: International conference on acoustics, speech, and signal processing, pp 381–384. 10.1109/ICASSP.1990.115702

[CR39] Komi PV (1984). Physiological and biomechanical correlates of muscle function: effects of muscle structure and stretch-shortening cycle on force and speed. Exerc Sport Sci Rev.

[CR40] Kubo K, Kanehisa H, Kawakami Y, Fukunaga T (2001). Influences of repetitive muscle contractions with different modes on tendon elasticity in vivo. J Appl Physiol (1985).

[CR41] Liu JZ, Shan ZY, Zhang LD, Sahgal V, Brown RW, Yue GH (2003). Human brain activation during sustained and intermittent submaximal fatigue muscle contractions: an FMRI study. J Neurophysiol.

[CR42] MacIntosh BR, Herzog W, Suter E, Wiley JP, Sokolosky J (1993). Human skeletal muscle fibre types and force: velocity properties. Eur J Appl Physiol Occup Physiol.

[CR43] MacIntosh BR, Holash RJ, Renaud JM (2012). Skeletal muscle fatigue-regulation of excitation-contraction coupling to avoid metabolic catastrophe. J Cell Sci.

[CR44] MacNaughton MB, MacIntosh BR (2006). Reports of the length dependence of fatigue are greatly exaggerated. J Appl Physiol (1985).

[CR45] Maffiuletti NA, Aagaard P, Blazevich AJ, Folland J, Tillin N, Duchateau J (2016). Rate of force development: physiological and methodological considerations. Eur J Appl Physiol.

[CR46] McNair PJ, Depledge J, Brettkelly M, Stanley SN (1996). Verbal encouragement: effects on maximum effort voluntary muscle action. Br J Sports Med.

[CR47] Merletti R, Knaflitz M, De Luca CJ (1990). Myoelectric manifestations of fatigue in voluntary and electrically elicited contractions. J Appl Physiol (1985).

[CR48] Milner-Brown HS, Stein RB, Yemm R (1973). The orderly recruitment of human motor units during voluntary isometric contractions. J Physiol.

[CR49] Morel B, Clémençon M, Rota S, Millet GY, Bishop DJ, Brosseau O, Rouffet DM, Hautier CA (2015). Contraction velocity influence the magnitude and etiology of neuromuscular fatigue during repeated maximal contractions. Scand J Med Sci Sports.

[CR50] Mosso A (1906). Fatigue (Translated by Drummond, M., Drummond, W. B.).

[CR51] Noakes TD (2012). Fatigue is a brain-derived emotion that regulates the exercise behavior to ensure the protection of whole body homeostasis. Front Physiol.

[CR52] Noakes TD, St Clair Gibson A, Lambert EV (2005). From catastrophe to complexity: a novel model of integrative central neural regulation of effort and fatigue during exercise in humans: summary and conclusions. Br J Sports Med.

[CR53] Penasso H, Thaller S (2017). Determination of individual knee-extensor properties from leg-extensions and parameter identification. Math Comput Model Dyn Syst.

[CR54] Place N, Yamada T, Bruton JD, Westerblad HK (2010). Muscle fatigue: from observations in humans to underlying mechanisms studied in intact single muscle fibres. Eur J Appl Physiol.

[CR55] Rockenfeller R, Günther M, Schmitt S (2015). Götz T (2015) Comparative sensitivity analysis of muscle activation dynamics. Comput Math Methods Med.

[CR56] Rode C, Siebert T, Blickhan R (2009). Titin-induced force enhancement and force depression: a ‘sticky-spring’ mechanism in muscle contractions?. J Theor Biol.

[CR57] Rzanny R, Stutzig N, Hiepe P, Gussew A, Thorhauer HA, Reichenbach JR (2016). The reproducibility of different metabolic markers for muscle fiber type distributions investigated by functional 31P-MRS during dynamic exercise. Z Med Phys.

[CR58] Sale DG (2002). Postactivation potentiation: role in human performance. Exerc Sport Sci Rev.

[CR59] Shorten PR, O’Callaghan P, Davidson JB, Soboleva TK (2007). A mathematical model of fatigue in skeletal muscle force contraction. J Muscle Res Cell Motil.

[CR60] Sidhu SK, Cresswell AG, Carroll TJ (2013). Corticospinal responses to sustained locomotor exercises: moving beyond single-joint studies of central fatigue. Sports Med.

[CR61] Siebert T, Sust M, Thaller S, Tilp M, Wagner H (2007). An improved method to determine neuromuscular properties using force laws—from single muscle to applications in human movements. Hum Mov Sci.

[CR62] Siebert T, Rode C, Herzog W, Till O, Blickhan R (2008). Nonlinearities make a difference: comparison of two common Hill-type models with real muscle. Biol Cybern.

[CR63] Solnik S, Rider P, Steinweg K, DeVita P, Hortobágyi T (2010). Teager-Kaiser energy operator signal conditioning improves EMG onset detection. Eur J Appl Physiol.

[CR64] Stutzig N, Siebert T (2015). Muscle force compensation among synergistic muscles after fatigue of a single muscle. Hum Mov Sci.

[CR65] Stutzig N, Rzanny R, Moll K, Gussew A, Reichenbach JR, Siebert T (2017). The pH heterogeneity in human calf muscle during neuromuscular electrical stimulation. Magn Reson Med Sci.

[CR66] Sust M, Schmalz T, Beyer L, Rost R, Hansen E, Weiss T (1997). Assessment of isometric contractions performed with maximal subjective effort: corresponding results for EEG changes and force measurements. Int J Neurosci.

[CR67] Sust M, Schmalz T, Linnenbecker S (1997). Relationship between distribution of muscle fibres and invariables of motion. Hum Mov Sci.

[CR68] Taylor JL, Amann M, Duchateau J, Meeusen R, Rice CL (2016). Neural contributions to muscle fatigue: from the brain to the muscle and back again. Med Sci Sports Exerc.

[CR69] Thaller S, Wagner H (2004). The relation between Hill’s equation and individual muscle properties. J Theor Biol.

[CR70] Thaller S, Tilp M, Sust M (2010). The effect of individual neuromuscular properties on performance in sports. Math Comput Model Dyn Syst.

[CR71] Till O, Siebert T, Blickhan R (2014). Force depression decays during shortening in the medial gastrocnemius of the rat. J Biomech.

[CR72] Van Eijden T, Kouwenhoven E, Verburg J, Weijs W (1986). A mathematical model of the patellofemoral joint. J Biomech.

[CR73] Van Soest AJ, Bobbert MF (1993). The contribution of muscle properties in the control of explosive movements. Biol Cybern.

[CR74] Von Tscharner V (2000). Intensity analysis in time-frequency space of surface myoelectric signals by wavelets of specified resolution. J Electromyogr Kinesiol.

[CR75] Wagner H, Thaller S, Dahse R, Sust M (2006). Biomechanical muscle properties and angiotensin-converting enzyme gene polymorphism: a model-based study. Eur J Appl Physiol.

[CR76] Westad C, Westgaard RH, De Luca CJ (2003). Motor unit recruitment and derecruitment induced by brief increase in contraction amplitude of the human trapezius muscle. J Physiol.

[CR77] Westerblad HK, Allen DG (1991). Changes of myoplasmic calcium concentration during fatigue in single mouse muscle fibers. J Gen Physiol.

[CR78] Williams CA, Ratel S (2009) Human muscle fatigue. Routledge, London. 10.4324/9780203885482

